# Psychometric validation of the Chinese version of the PROMIS-29 profile in community-dwelling older adults with multimorbidities

**DOI:** 10.3389/fpubh.2025.1631442

**Published:** 2025-10-28

**Authors:** Ting Zhao, Yan Zhang, Qinghua Cui, Min Zhang, Xiaoxia Han, Jialin Chen

**Affiliations:** 1 School of Nursing and Health, Zhengzhou University, Zhengzhou, Henan, China; 2 The First Affiliated Hospital of Zhengzhou University, Zhengzhou, Henan, China; 3 The Fifth Affiliated Hospital of Zhengzhou University, Zhengzhou, Henan, China; 4 School of Nursing, Fudan University, Shanghai, China

**Keywords:** PROMIS-29, psychometric validation, multimorbidity, older adults, item response theory

## Abstract

**Background:**

The Patient-Reported Outcomes Measurement Information System 29-item (PROMIS-29) profile is commonly used to measure patients’ self-reported health status. This study examined the psychometric properties of the Chinese version of the PROMIS-29 (v2.1) in older individuals.

**Methods:**

Cognitive interviews and psychometric evaluations were conducted between January and August 2023. Cognitive interviews were conducted in accordance with the Cognitive Interviewing–Reporting Framework. The Chinese version of the PROMIS-29 was revised based on feedback from respondents and experts. The structural validity of the PROMIS-29 was evaluated using confirmatory factor analysis (CFA). The convergent and discriminant validities were assessed by calculating Spearman’s rank correlation and comparing known group differences. Reliability was assessed with Cronbach’s *α*, and item response theory (IRT)-based psychometric assessment was performed using Rasch models for unidimensionality, local independence, item characteristic curve (ICC) matrices, and model fit. Differential item functioning (DIF) was used to examine demographic bias.

**Results:**

A total of 606 cases with a mean age of (72.88 ± 7.2) were included. The CFA showed acceptable convergence. PROMIS-29 was significantly correlated with comparable domains in the legacy questionnaires. Cronbach’s *α* of the instrument ranged from 0.82 to 0.95. The Rasch models explained 44.5 and 73.2% of the variance, respectively. Local independence analysis showed that the maximum standardized residual correlation coefficients between items within the short form ranged from 0 to 0.69 in absolute value. All items demonstrated excellent discriminatory power. A good Rasch model fit was revealed in terms of the outfit MNSQ, infit MNSQ, and overall outfit MNSQ. Most items showed acceptable item characteristic curve matrices and did not have statistically significant bias (DIF).

**Conclusion:**

The Chinese version of the PROMIS-29 showed acceptable psychometric properties in community-dwelling older adults with multimorbidities. These findings suggest that this questionnaire can be beneficial in the assessment of symptoms and function in the older population.

## 1 Introduction

With the global population aging, multimorbidity among older adults is an increasing concern worldwide (1). The American Geriatrics Society Expert Panel developed a guiding principle for the care of older adults with multimorbidity, which suggests the importance of patient perspective for clinical decision making, health research, and policy making (2, 3). Information collected directly from patients could provide authentic and reliable support for primary care workers to make medical decisions and manage disease, as well as provide evidence for mitigating adverse outcomes, such as disability and debilitation, in community-dwelling older adults with multimorbidities (4). Patient-reported outcomes (PROs) are assessments of patients’ health status reported directly by patients, without interpretation by healthcare professionals or others (5). PROs are also associated with physical and psychological health-related quality of life (HRQoL) (6). Community-dwelling older adults with chronic diseases often experience several psychological, physical, and social dysfunctions (7). Accurate measurements of PROs can provide important information to assist in the education and self-management of community-dwelling older adults with multimorbidities, which remain to be investigated.

Recently, the Patient Reported Outcomes Measurement Information System (PROMIS) has attracted attention as a new and efficient instrument for evaluating person-centered health. The PROMIS 29-item profile (PROMIS-29 v2.1) was developed using an item response theory, calibrated, and scored based on contemporary samples. The PROMIS-29 has an advantage over the HRQOL tools, as it measures a more comprehensive range of symptoms and functions (8). It covers commonly reported health and functional problems, such as physical function, pain interference, anxiety, depression, fatigue, sleep disturbances, and the ability to participate in social roles and activities (3). Owing to its brevity and breadth, the PROMIS-29 has been used widely in community and clinical samples, such as in those with chronic pulmonary diseases, hemophilia, chronic lower back pain, and multiple chronic conditions (8–12). Robust psychometric properties have been set up in these contexts. PROMIS-29 was translated from English into simplified Chinese by the members of PROMIS National Center China (PNC-China) strictly following the Functional Assessment of Chronic Illness Therapy translation method (13, 14).

However, research on the use of PROMIS tools in community-dwelling older adults with multimorbidities is limited. Further, PROMIS-29 v2.1 has not been validated or applied in Chinese community-dwelling older adults with multimorbidities. The measurement domains of the PROMIS-29 include the most commonly reported health and functional issues among older adults with chronic conditions, which may offer a relatively comprehensive self-assessment of health status (15). From this perspective, the PROMIS-29 may be a promising tool for symptom management in primary healthcare and clinical research in community-dwelling older adults with multimorbidities. The purpose of this study was to conduct cognitive interviews using a simplified Chinese version of the PROMIS-29 and perform a psychometric evaluation on a sample of community-dwelling older adults with multimorbidities in mainland China.

## 2 Materials and methods

### 2.1 Study design and settings

This was a cross-sectional study. Two phases were conducted: cognitive interviews and psychometric evaluation of the Chinese version of the PROMIS-29 v2.1 scale.

### 2.2 Phase I: cognitive interviews

Cognitive interviews were conducted in strict accordance with the cognitive interviewing reporting framework (CIRF) before the psychometric evaluation (16). There were 25 community older adults with multimorbidities from Zhengzhou City (including 13 men and 12 women aged 62–90 years) who were interviewed. The primary technique used was verbal probing and thinking out loud. The interview materials were coded using the Question Appraisal System (QAS-99) evaluation system (17). Twenty respondents were included in the first round of cognitive interviews. Most respondents found that the content of the scale, including guidelines, number of items, and fonts, was well designed and easy to understand. Some respondents raised doubts about 12 of the items. A total of six items focused on “clarification,” which were related to grammar and word usage. For example, responses to four items in the domain of the ability to participate in social roles and activities were difficult for respondents. The aged tended to ignore the negative meaning of “难以完成” of the questions in their answers, showing significant logical errors. Therefore, we changed the original negative sentence to an affirmative sentence while reversing the scores of the options in which the semantic context of the options matched, keeping the logical relationship between the scores and concepts of the domain unchanged. In addition, three items related to “knowledge/memory,” including words, and two items that were difficult to understand were selected due to different socio-cultural backgrounds. The Chinese version of the PROMIS-29 was revised based on feedback from respondents and experts. Most items were easy to understand and conformed to the culture. Five respondents in the second round did not raise any questions, and they completed the questionnaire easily within 10 min or less. Thus, the scale was ready for psychometric testing.

### 2.3 Phase II: psychometric evaluation

#### 2.3.1 Sampling

Eligible community-dwelling older adults were selected for this study using convenience sampling. Patients were recruited for the study if they met the following criteria: (1) The presence of two or more of the 18 common geriatric chronic diseases included in the functional comorbidity index (FCI) scale (18), (2) age ≥ 60 years, (3) permanent residents of the community who have lived in the community for more than 6 months, and (4) those who provided voluntary informed consent. The exclusion criteria included the following: (1) severe disease and inability to cooperate with the study and (2) cognitive dysfunction or severe mental disorder.

#### 2.3.2 Measures

##### 2.3.2.1 Sociodemographic information questionnaire

A sociodemographic information questionnaire was developed to collect sociodemographic and clinical data regarding gender, age, marital status, education level, pre-retirement/current employment, co-residence, health insurance, smoking, alcohol consumption, physical activity, and number of chronic diseases. The number of chronic diseases was determined using the FCI index. The FCI scale was developed by Groll et al. (18) in 2005 to assess comorbidity status in the aged. These included degenerative disc disease, arthritis, asthma, osteoporosis, diabetes, angina pectoris, chronic obstructive pulmonary disease/respiratory distress syndrome/emphysema, heart attack/myocardial infarction, congestive heart failure, heart attack, stroke/transient ischemic attack, neurological diseases, peripheral vascular diseases, upper digestive tract diseases, depression, anxiety/panic disorder, hearing impairment, visual impairment, and obesity (BMI > 30 kg/m^2^). Each type of disease scored 1 point; no disease scored 0 points; the scores were added to the FCI index. The FCI checklist also includes an additional item for manually adding chronic diseases that patients may have other than the 18 mentioned above, which will be included in the final score. A higher score indicates several comorbidities. Sociodemographic data were obtained from electronic health records by trained nursing researchers. Clinical data were collected, in part, from electronic health records and supplemented with self-reported patient data.

##### 2.3.2.2 The patient-reported outcomes measurement information system 29-item profile (PROMIS-29)

PROMIS-29 v2.1 comprises 29 items in seven domains: physical function, anxiety, depression, fatigue, sleep disturbance, ability to participate in social roles and activities, pain interference, and pain intensity. We used a 5-point Likert scale (range: 1–5) to measure the severity or frequency of symptoms. The single pain intensity items were scored separately with a response scale ranging from 0 (no pain) to 10 (most severe pain imaginable) (9). Item scores in each domain were summarized and converted into a T-score metric, with values of 50 (SD = 10) representing a mean of the U. S. general population^1^. A higher score implied greater amplitude of the measured concept (19).

To evaluate the PROMIS-29 tool as a HRQOL instrument that can be used to assess the quality of life and as a symptom instrument for comprehensive assessment of health status, the convergent validity of the PROMIS-29 was tested using the Short Form-12 Health Survey (SF-12), the eight-item Somatic Symptom Scale (SSS-8), Patient Health Questionnaire-2 (PHQ-2), and the Generalized Anxiety Disorder-2 (GAD-2) (20–26).

#### 2.3.3 Data collection

The survey was conducted between January and August 2023. Recruitment of the study population was done during a visit to the family doctor, and informed consent was obtained. We also designed a WeChat mini program suitable for the aging population who can use smartphones. Age-friendly design has been applied to this mini-program. For example, the font of the page was enlarged, and each question and option of the scale was set up with a voice assistant to minimize difficulties for the aged in filling in the questionnaire. We tested the feasibility of the WeChat applet based on the ISO 9241-11:2018 usability framework (27). Older adults could choose paper or electronic questionnaires according to their preferences. For older adults who were unable to complete the questionnaire by themselves (such as those with visual impairments), the researcher relayed the questionnaire content verbally without further explanation. Patients were informed confidentiality of the research.

#### 2.3.4 Statistical analysis

Descriptive statistics were calculated for sample characteristics and study variables. Continuous variables are presented by means and standard deviations, while categorical variables are represented by counts and percentages. Floor or ceiling effects were considered noteworthy, which were defined as greater than 20% of the response for the proportion of minimum or maximum, respectively (28).

Confirmatory factor analysis (CFA) was performed for the structural validity of the PROMIS-29. The fitness of the proposed seven-factor model to the data was evaluated using the comparative fit index (CFI), χ^2^/degree of freedom (χ^2^/df), standardized root-mean-squared residual (SRMR), root mean square error of approximation (RMSEA), and Akaike’s information criterion (AIC). A CFI > 0.90, χ^2^/df < 3, and SRMR < 0.05 indicated a good fit (29, 30). RMSEA < 0.05 indicated a good fit, and < 0.08 was acceptable (31). The smaller the AIC value, the better the model fit. Items with factor loadings equal to or higher than the standard of 0.4 were retained (32).

Regarding structural validity, known-group validity was evaluated for groups with different expected scores: gender, age group (compared with three age groups), and the number of chronic illnesses (FCI). These groups were picked in view of a literature review and the authors’ clinical judgment (10). The Mann–Whitney U test was used for comparisons between two groups, and the Kruskal–Wallis H rank sum test was used for comparisons between multiple groups.

Due to the non-normality of the sample, Spearman’s rank correlations between scores of the PROMIS-29 and their corresponding legacy PRO measures were calculated to evaluate convergent validity. Correlation coefficients of 0.9–1.0 indicate very strong, 0.7–0.89 strong, 0.5–0.69 moderate, and 0.3–0.49 weak relationships (11). The correlations between the PROMIS-29 domain scores and dissimilar constructs of legacy measures supported discriminant validity; these correlations were expected to be less than 0.60 (11). The reliability of the measures was evaluated by Cronbach’s *α* coefficient and split-half reliability. Cronbach’α of 0.6–0.7 indicated acceptable levels, and ≥ 0.8 indicated very good reliability levels (33).

IRT-based psychometric assessment was done using Rasch models for unidimensionality, local independence, ICC matrices, and model fit. The absolute values of the maximum standardized residual correlation coefficients < 0.7 were considered to have good local independence (34). Generally, more than 40% of the variance explained by the Rasch model indicated unidimensionality of the domain (35). The fit of each item to the Rasch model was evaluated using infit and outfit mean-square statistics (MNSQ). Similarly, the fit of each domain was evaluated based on the overall outfit MNSQ. Av value of 0.5 to 1.5 is generally regarded as indicative of a satisfactory fit for outfit MNSQ, infit MNSQ, and overall outfit MNSQ values (36). Point-measure correlation (PTMEA Corr.) is an indicator of item discrimination, with values greater than 0.40 considered acceptable, indicating that the item positively correlates with the underlying latent trait and effectively discriminates between respondents of different ability levels (36).

To check whether the measures delivered biased results across various populations, the differential item functioning (DIF) of each item was checked on the PROMIS-29 scale. DIF analyses were performed to test measurement invariance and determine whether the probability of patients with the same characteristics from different groups responding to certain items is different (37). Items with significant DIF values indicated measurement bias; differences of ≥ 0.5 logits in item difficulties were considered meaningful (38). No DIF was expected for any of the variables since the universal usability of the PROMIS-29. Accordingly, measurement invariance was evaluated by pondering DIF of the PROMIS social function short forms in view of age, gender, and education.

Statistical significance was set at *p* value < 0.05. Data were analyzed using SPSS27.0, Amos Graphics24.0, and Winsteps3.72.0.

## 3 Results

### 3.1 Descriptive statistics

A total of 628 patients were surveyed in this study; 22 questionnaires with missing information were excluded and 606 valid questionnaires were analyzed. The mean age of the participants was (72.88 ± 7.2) years. In the cohort, 25.6% of the patients had attained high school and equivalent education. Most of the participants were married (74.8%), and most patients were employed as workers pre-retirement (33.7%) and staff of public institutions (29.4%). The mean FCI score of this multiple chronic disease group was 3.25. Most patients had healthy lifestyles and did not smoke or drink alcohol (Table 1).

**Table 1:** Characteristics of the study patients (*N* = 606).

Characteristic		M ± SD / *N* (%)
Age		72.88 ± 7.2
Sex	Male	277 (45.7)
	Female	329 (54.3)
Education level	Primary school or below	186 (30.7)
	Junior high school	176 (29.0)
	Senior high school or equivalent	155 (25.6)
	College level or higher	89 (14.7)
Marital status	Single, divorced, widowed	153 (25.2)
	Married	453 (74.8)
Preretirement/ current occupation	Worker	204 (33.7)
	Staff of public institutions	178 (29.4)
	Farmer	166 (27.4)
	Business, services, etc	58 (9.6)
Living style	Living with families or friends	543 (89.6)
	Living alone	63 (10.4)
Personal monthly income	≤2000 yuan	225 (37.1)
	2001–3,000 yuan	73 (12.0)
	3,001–4,000 yuan	145 (23.9)
	4,001–5,000 yuan	98 (16.2)
	>5,000 yuan	65 (10.7)
Medical insurance	Employee health insurance	370 (61.1)
	Resident medical insurance	59 (9.7)
	Rural health insurance	166 (27.4)
	Without health insurance	11 (1.8)
Smoking	No or quitting	535 (88.3)
	Yes	71 (11.7)
Drinking alcohol	No or quitting	516 (85.1)
	Yes	90 (14.9)
Exercise	≥3 times a week	413 (68.2)
	Once or twice a week	124 (20.5)
	Never	69 (11.4)
FCI score		3.25 (1.6)
Questionnaire form	Paper version	488 (80.5)
	WeChat mini program	118 (19.5)

Descriptive statistics of the patients are presented in Table 2. There were significant ceiling effects for physical function (44.4%) and floor effects for pain interference (41.3%). Except for these, no ceiling or floor effects were observed for any of the other short forms. The mean T-scores for each PROMIS-29 domain are shown in Table 2.

**Table 2:** T-scores and floor and ceiling effects of the PROMIS-29 (*N* = 606).

Domain	Mean	MD	Floor *n* (%)	Ceiling *n* (%)
Physical function	48.9	8.7	0.2	44.4
Anxiety	48.7	8.7	0.2	0.5
Depression	48.4	8.1	0.2	0.2
Fatigue	46.0	9.5	0.2	0.3
Sleep disturbance	50.7	8.7	4.1	0.5
Social domain	56.5	8.5	1.3	1.0
Pain interference	50.3	8.4	41.3	0.3

Social domain = ability to participate in social roles and activities.

### 3.2 Structural validity

**Figure 1:**
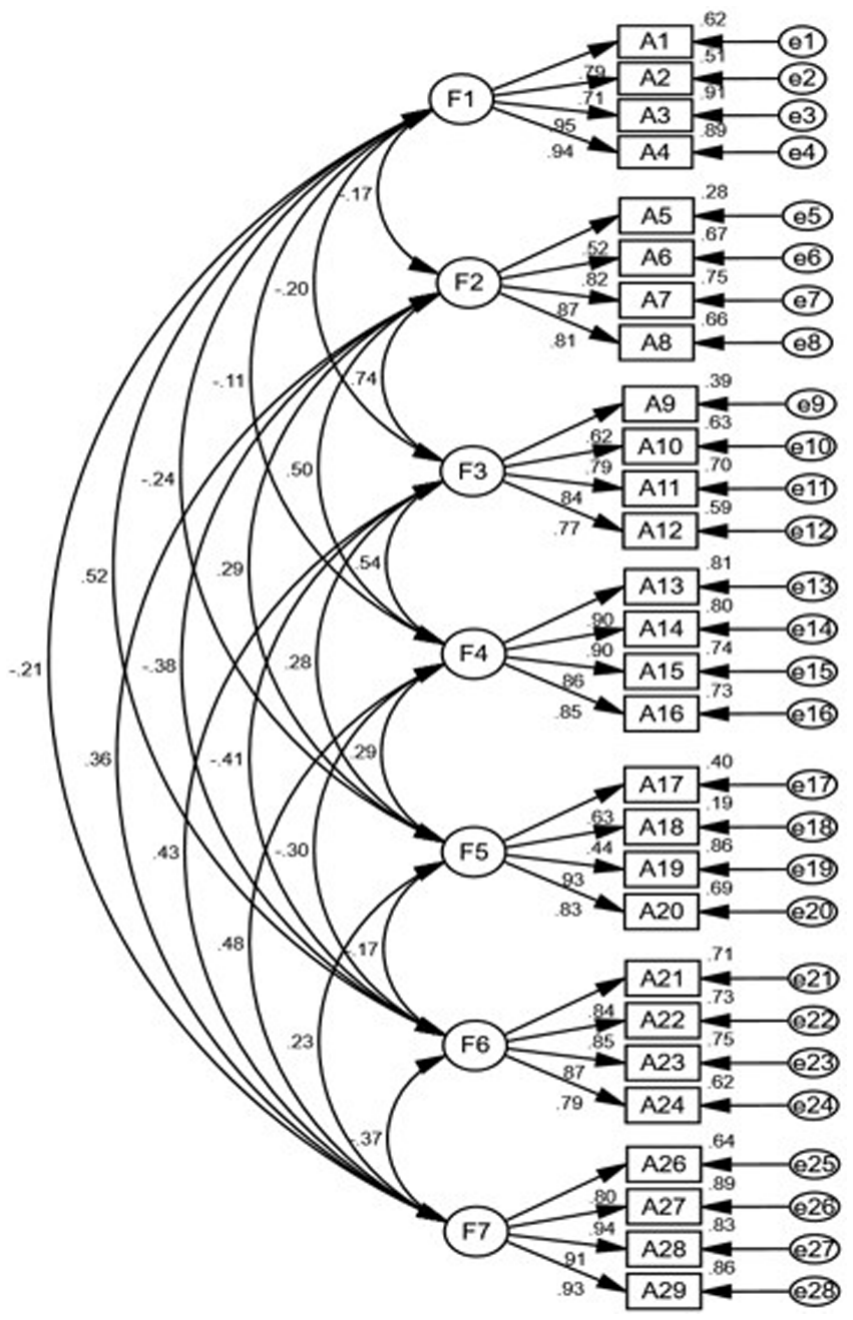
Confirmatory factor analysis for the seven-factor model of the PROMIS-29. F1–F7: physical function, anxiety, depression, fatigue, sleep disturbances, ability to participate in social roles and activities, and pain interference, respectively.

The goodness-of-fit indices and the main model fit results of the CFA are presented in Table 3 and Figure 1. The original seven-factor model structure within the PROMIS-29 was confirmed based on the following statistics (CFI = 0.95, SRMR = 0.05, RMSEA = 0.05, χ^2^/df = 2.85). The standardized regression coefficients were 0.438–0.954, indicating acceptable structural validity of the short form for each domain.

**Table 3:** Scores and fit indices of CFA and internal consistency for PROMIS-29 domains (*N* = 606).

Model	CFI	AIC	RMSEA (95%)	SRMR	χ^2^/df	Cronbachs’ α	Split-half coefficient
Seven-factor model	0.95	1090.63	0.05	0.05	2.85		
Single-factor model							
Physical function	0.99	36.58	0.17 (0.713 ~ 0.954)	0.01	18.6	0.92	0.86
Anxiety	0.99	26.58	0.08 (0.525 ~ 0.866)	0.02	5.29	0.83	0.81
Depression	0.99	31.89	0.11 (0.621 ~ 0.838)	0.02	7.94	0.82	0.79
Fatigue	0.97	88.94	0.24 (0.852 ~ 0.899)	0.03	36.47	0.93	0.90
Sleep disturbance	0.95	70.7	0.21 (0.438 ~ 0.927)	0.06	27.36	0.80	0.69
Ability to participate in social roles and activities	0.99	37.77	0.13(0.790 ~ 0.869)	0.02	10.89	0.90	0.89
Pain interference	0.99	37.51	0.13 (0.802 ~ 0.941)	0.01	10.76	0.94	0.94

### 3.3 Convergent validity and discriminant validity

As shown in Table 4, the correlations between most PROMIS-29 domains and comparable PRO measures were significantly strong (r > 0.60). The sleep disturbance domain was strongly correlated with the SSS-8 domain of sleep trouble, with a Spearman coefficient of 0.824. Thus, convergent validity was achieved.

**Table 4:** Spearman’s coefficients within the PROMIS-29 scales and legacy PRO domains (*N* = 338).

Domain	Physical function	Anxiety	Depression	Fatigue	Sleep disturbance	Social domain	Pain interference
Physical function		−0.325*	−0.344*	−0.322*	−0.228*	0.558*	−0.313*
Anxiety	−0.325*		0.602*	0.419*	0.412*	−0.387*	0.338*
depression	−0.344*	0.602*		0.373*	0.332*	−0.384*	0.377*
Fatigue	−0.322*	0.419*	0.373*		0.362*	−0.269*	0.406*
Sleep disturbance	−0.228*	0.412*	0.332*	0.362*		−0.230*	0.351*
Social domain	0.558*	−0.387*	−0.384*	−0.269*	−0.230*		−0.302*
Pain interference	−0.313*	0.338*	0.377*	0.406*	0.351*	−0.302*	
PCS	0.611*	−0.340*	−0.356*	−0.363*	−0.318*	0.512*	−0.614*
MCS	0.141*	−0.490*	−0.458*	−0.394*	−0.406*	0.241*	−0.292*
GAD-2	−0.300*	0.776*	0.565*	0.467*	0.425*	−0.405*	0.419*
PHQ-2	−0.348*	0.551*	0.619*	0.448*	0.437*	−0.405*	0.401*
SSS-8 pain	−0.290 *	0.340*	0.263*	0.339*	0.284*	−0.216*	0.699*
SSS-8 tiredness	−0.404*	0.481*	0.400*	0.618*	0.403*	−0.333*	0.402*
SSS-8sleep trouble	−0.223*	0.430*	0.346*	0.401*	0.824*	−0.193*	0.349*
SF-12 PF	0.680*	−0.321*	−0.355*	−0.277*	−0.273*	0.572*	−0.446*
SF-12 SF	0.414*	−0.498*	−0.500*	−0.454*	−0.417*	0.503*	−0.469*

Social domain = ability to participate in social roles and activities.

*p < 0.01.

However, correlations between domains of PROMIS-29 and its conceptually different legacy PRO measures or domains of PROMIS-29 were not significantly correlated (r < 0.5). For example, the PROMIS-29 physical function score correlated with GAD-2 and PHQ-2 to a low degree (r < 0.4), and between PROMIS-29 anxiety and depression and SF-12 PF. Inter-factor correlations were weaker for most PROMIS-29 domains. A weak correlation was seen between the scores for PROMIS-29 domains related to “ability to participate in social roles and activities” and “sleep disturbance” (r = 0.230, *p* < 0.01), which supported satisfactory discriminant validity (Table 4).

To examine the known-group validity of PROMIS-29, the scores were compared across patients of different genders, ages, and number of chronic illness (FCI) statuses. Compared to men, women had higher T-scores for anxiety, depression, fatigue, sleep disturbance, and pain interference, and lower T-scores for their ability to participate in social roles and activities. With increasing age, the T-score for physical function and the ability to play social roles and participate in social activities decreased. The FCI scores were significantly different in all seven domains of PROMIS-29, with populations with FCI scores ≥ 4 tending to have more terrible health conditions than those with FCI scores ≤ 3. This indicates that the Chinese version of PROMIS-29 can generally distinguish between different groups and has acceptable known-group validity (Additional File 1).

### 3.4 Reliability

For the reliability analysis, the internal consistency coefficients and Guttman split-half coefficients were calculated. The Cronbach’s *α* values and split-half coefficients of the PROMIS-29 were above the standard of 0.70 (Table 3).

### 3.5 Item-level psychometric properties (Rasch analysis)

The item-level statistics from the Rasch analysis are presented in Table 5. All items demonstrated excellent discriminatory power, with Point-Measure Corr. values ranging from 0.61 to 0.94, well above the 0.40 threshold. All the items except Item Numbers “B1” and “G1” showed fit statistics ranging from 0.5 to 1.5, which was an acceptable fit. In addition, the items were located across a wide range of difficulties, ranging from −1.63 to +1.76 logits.

**Table 5:** Item fit and item discrimination analysis in the PROMIS-29.

Domain	Item number	Difficulty	Model S. E.	Infit MNSQ	Outfit MNSQ	Overall Infit MNSQ	PTMEA Corr.
Physical function	A1	1.14	0.09	1.15	1.06	1.02	0.87
	A2	1.76	0.09	0.86	0.81		0.89
	A3	−1.06	0.14	1.24	0.80		0.80
	A4	−1.84	0.17	0.83	1.04		0.77
Anxiety	B1	−0.02	0.11	1.82	1.72	0.99	0.70
	B2	−0.21	0.10	0.67	0.69		0.85
	B3	0.24	0.11	0.65	0.64		0.82
	B4	−0.01	0.11	0.84	0.87		0.81
Depression	C1	−1.36	0.10	1.37	1.42	0.95	0.84
	C2	−0.06	0.11	0.82	0.85		0.84
	C3	0.21	0.11	0.75	0.83		0.83
	C4	1.21	0.13	0.86	0.65		0.72
Fatigue	D1	−1.13	0.15	0.91	0.91	0.96	0.94
	D2	0.18	0.16	1.01	1.07		0.93
	D3	0.60	0.15	0.74	0.69		0.93
	D4	0.35	0.16	1.20	1.20		0.90
Sleep disturbance	E1	−0.88	0.08	0.68	0.73	0.99	0.86
	E2	0.39	0.08	1.37	1.40		0.72
	E3	0.80	0.09	0.59	0.56		0.89
	E4	0.46	0.08	1.33	1.21		0.83
Social roles	F1	0.51	0.15	1.03	1.06	0.99	0.93
	F2	−0.06	0.15	0.92	0.92		0.93
	F3	0.69	0.16	0.61	0.56		0.93
	F4	0.24	0.15	1.40	1.38		0.90
Pain interference	G1	−1.63	0.14	1.48	1.73	0.97	0.89
	G2	−0.03	0.15	0.87	0.81		0.94
	G3	0.91	0.15	0.73	0.63		0.93
	G4	0.75	0.15	0.80	0.70		0.93
Pain intensity	F1	−0.26	0.04	0.86	0.85		0.61

Social roles: ability to participate in social roles and activities.

### 3.6 Unidimensionality

As shown in Table 6, the Rasch model explained more than 40% of the variance (ranging from 47.8 to 71.5%). The number of characteristic roots in the first control was less than 3 (ranging from 1.5 to 2.5). Thus, the Rasch model analysis illustrated the unidimensionality of the domains from a side perspective.

**Table 6:** Unidimensionality results.

Domain	Rasch model explanatory quantity	The characteristic root value of the first control
Physical function	71.5%	1.9
Anxiety	47.8%	1.7
Depression	61.0%	1.7
Fatigue	70.0%	1.9
Sleep disturbance	48.6%	2.5
Social domain	59.0%	1.5
Pain interference	85.6%	1.8

Social domain = ability to participate in social roles and activities.

### 3.7 Local Independence and monotone increasing hypothesis

**Figure 2:**
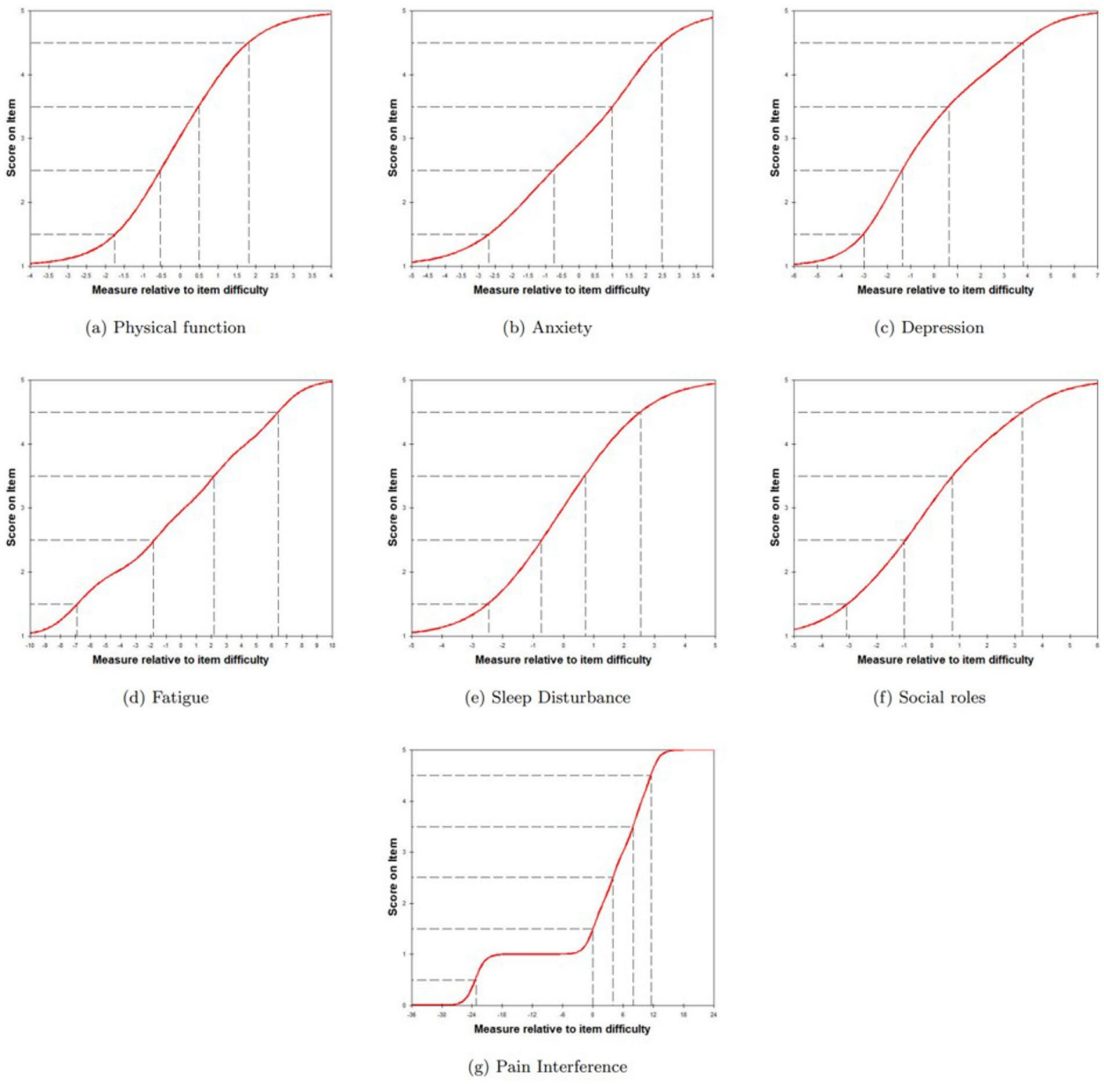
Characteristic item curves for each PROMIS-29 domain.

The absolute values of the maximum standardized residual correlation coefficients between the items within each short form ranged from 0 to 0.69. All seven short forms were considered to have good local independence. The item characteristic curves satisfy the assumption of monotonic progressivity (Figure 2).

### 3.8 Measurement invariance

Measurement invariance was evaluated by examining the DIF. The results showed that item 2 “my sleep was refreshing” and item 4 “I had difficulty falling asleep” of sleep disturbance had functional differences across age (<60 years old and 70–79 years old), indicating that there is cross-age measurement inequivalence in these items. Except for these two items, no significant DIF existed in the other PROMIS-29 items in patients with different backgrounds (gender, age, and education), suggesting that the instrument provided unbiased results in this population overall (Tables 7–9).

**Table 7:** Gender-based differences in DIF of the PROMIS-29 questionnaire.

Domain	Item	Male	Female	DIFΔ	*p*
Physical function	A1	1.01	1.27	−0.26	0.16
	A2	1.86	1.64	0.22	0.22
	A3	−1.06	−1.06	0.00	1.00
	A4	−1.81	−1.88	0.07	0.84
Anxiety	B1	0.01	−0.05	0.06	0.79
	B2	−0.11	−0.28	0.17	0.43
	B3	0.04	0.42	−0.38	0.09
	B4	0.06	−0.07	0.13	0.55
Depression	C1	−1.53	−1.24	0.29	0.17
	C2	0.02	−0.12	0.14	0.52
	C3	0.34	0.13	0.22	0.34
	C4	1.19	1.24	−0.05	0.86
Fatigue	D1	−1.21	−1.05	−0.15	0.61
	D2	0.18	0.18	0.00	1.00
	D3	0.52	0.67	−0.15	0.63
	D4	0.52	0.19	0.33	0.29
Sleep disturbance	E1	−0.93	−0.81	−0.13	0.44
	E2	−0.58	−0.17	−0.41	0.06
	E3	0.96	0.65	0.31	0.07
	E4	0.60	0.32	0.27	0.11
Social domain	F1	0.27	0.67	−0.41	0.18
	F2	0.10	−0.18	0.28	0.37
	F3	−0.62	−0.74	0.12	0.71
	F4	0.27	0.22	0.05	0.88
Pain interference	G1	−1.86	−1.45	−0.40	0.17
	G2	−0.03	−0.03	0.00	1.00
	G3	0.95	0.91	0.03	0.91
	G4	1.00	0.57	0.44	0.16

Social domain = ability to participate in social roles and activities.

**Table 8:** Age-related comparisons in DIF of the PROMIS-29 questionnaire.

Domain	Item	~69(a)	70 ~ 79(b)	80 ~ (c)	DIFΔ/ P (a-b)	DIFΔ/ P (a-c)	DIFΔ/ P (b-c)
Physical function	A1	1.29	1.14	1.06	0.15/0.54	0.23/0.41	0.08/0.71
	A2	1.64	1.76	1.85	−0.12/0.60	−0.21/0.43	−0.09/0.67
	A3	−0.81	−1.12	−1.11	0.31/0.39	0.30/0.47	−0.01/0.97
	A4	−2.46	−1.72	−1.84	−0.74/0.18	−0.62/0.31	0.12/0.77
Anxiety	B1	0.27	−0.02	−0.34	0.29/0.25	0.61/0.05	0.31/0.24
	B2	−0.18	−0.21	−0.21	0.03/0.90	0.03/0.92	0.00/1.00
	B3	0.10	0.34	0.24	−0.24/0.36	−0.14/0.65	0.09/0.73
	B4	−0.18	−0.06	0.34	−0.12/0.61	−0.51/0.09	−0.39/0.16
Depression	C1	−0.91	−1.50	−1.55	0.58/0.024	0.64/0.04	0.21/0.84
	C2	−0.18	−0.10	0.14	−0.08/0.77	−0.32/0.31	−0.24/0.37
	C3	−0.13	0.28	0.42	−0.41/0.13	−0.55/0.08	−0.14/0.61
	C4	1.15	1.41	0.95	−0.26/0.42	0.21/0.56	0.46/0.14
Fatigue	D1	−1.46	−0.86	−1.28	−0.60/0.09	−0.19/0.66	0.41/0.29
	D2	0.01	0.22	0.35	−0.21/0.56	−0.34/0.45	−0.13/0.75
	D3	0.75	0.37	0.96	0.39/0.29	−0.21/0.64	−0.60/0.15
	D4	0.75	0.27	−0.01	0.48/0.19	0.76/0.09	0.28/0.49
Sleep disturbance	E1	−0.93	−0.90	−0.75	−0.03/0.86	−0.18/0.44	−0.15/0.49
	E2	−0.62	−0.22	−0.43	−0.40/0.03	−0.19/0.40	0.21/0.34
	E3	0.80	0.80	0.85	0.00/1.00	−0.05/0.84	−0.05/0.82
	E4	0.80	0.33	0.33	0.47/0.01	0.47/0.06	0.00/0.99
Social domain	F1	0.20	0.53	0.65	−0.33/0.43	−0.12/0.72	−0.12/0.55
	F2	0.08	−0.06	−0.13	0.14/0.74	0.20/0.66	0.07/0.85
	F3	−0.59	−0.59	−0.94	0.00/1.00	0.35/0.48	0.35/0.37
	F4	0.33	0.14	0.35	0.18/0.66	−0.02/0.96	−0.20/0.56
Pain interference	G1	−2.24	−1.41	−1.30	−0.82/0.01	−0.94/0.02	−0.11/0.76
	G2	−0.01	0.07	−0.26	−0.07/0.84	0.26/0.54	0.33/0.39
	G3	1.18	0.84	0.77	0.34/0.37	0.42/0.35	0.08/0.84
	G4	1.28	0.47	0.75	0.80/0.03	0.53/0.24	−0.27/0.49

Social domain = Ability to participate in social roles and activities.

(a) > 69 years, (b) 70 to 79 years, (c) ≥ 80 years.

**Table 9:** Education-level related comparisons in DIF of the PROMIS-29 questionnaire.

Domain	Item	Junior high school and below	Senior high school and above	DIFΔ	*p*
Physical function	A1	1.20	0.96	0.24	0.26
	A2	1.76	1.76	0.00	1.00
	A3	−1.06	−1.02	−0.03	0.92
	A4	−2.04	−1.35	−0.68	0.06
Anxiety	B1	0.08	−0.26	0.34	0.13
	B2	−0.23	−0.15	−0.08	0.74
	B3	0.17	0.45	−0.29	0.25
	B4	−0.01	−0.01	0.00	1.00
Depression	C1	−1.58	−0.65	0.92	0.00
	C2	−0.04	−0.12	0.08	0.75
	C3	0.35	−0.22	0.57	0.02
	C4	1.34	0.87	0.47	0.10
Fatigue	D1	−1.15	−1.08	−0.07	0.82
	D2	0.18	0.22	0.03	0.92
	D3	0.60	0.60	−0.00	1.00
	D4	0.39	0.29	0.10	0.77
Sleep disturbance	E1	−0.91	−0.82	−0.09	0.61
	E2	−0.27	−0.62	0.35	0.04
	E3	0.78	0.86	−0.08	0.66
	E4	0.40	0.60	−0.20	0.26
Social domain	F1	0.51	0.55	−0.04	0.91
	F2	−0.06	−0.11	0.05	0.90
	F3	−0.78	−0.41	−0.37	0.34
	F4	0.32	−0.01	0.32	0.37
Pain interference	G1	−1.60	−1.68	0.08	0.79
	G2	−0.03	0.02	−0.05	0.87
	G3	0.91	0.88	0.03	0.93
	G4	0.75	0.80	−0.05	0.87

Social domain = ability to participate in social roles and activities.

## 4 Discussion

The current study aimed to validate the PROMIS-29 profile in community-dwelling older adults with multimorbidities in mainland China. Unlike Huang’s study (24), which applied the Chinese version of PROMIS-29 to a postoperative population, this study examined the psychometric properties of the Chinese version of PROMIS-29 at a deeper micro level of a psychological measurement characteristic test. According to the PROMIS-29 guidelines and CIRF, a rigorous method was used to conduct cognitive interviews with the Chinese version of the PROMIS-29 questionnaire, which improved the accuracy and practicality of its application in community-dwelling older adults with multimorbidities.

No significant sensitivity bias was reported, such as “assumptions” and “sensitivity/bias” (13). However, the Chinese version of the PROMIS-29 would have comprehension bias when applied to the older population with chronic diseases due to the limitations of gender and age distribution. The Chinese version of the PROMIS-29 was designed for conceptual and semantic equivalence with the original version through cognitive interviews. Psychometric testing demonstrated that the measures reduced patient burden, along with sufficient reliability and construct validity in patients with multimorbidities.

Descriptive statistics showed that patients had lower scores for physical function, anxiety, depression, and fatigue, and higher scores for the ability to participate in social roles and activities, compared to the general population in the USA. The participants’ ability to participate in social roles and activities scored the highest of the seven health domains, similar to the results of studies by Kang and Ellen (9, 10). These results could be because the participants in the studies were outpatients or community residents. In addition, this score was higher than that of postoperative patients included in the research by Huang, and patients with breast cancer included in the study by Cai (13, 24), suggesting that community-dwelling older adults with multimorbidities might have better social function. However, the fatigue score of the participants was the lowest of the seven health domains, which was consistent with the situation of the complete sample in a Dutch study (10). The anxiety score was similar to that of a chronic population in a Dutch study (10), but the depression score was higher.

The present study found significant ceiling effects for physical function (44.4%) and floor effects for pain interference (41.3%). Similar results have been presented in previous studies (39, 40). Generally, the interference of the ceiling and floor effects should be considered according to the purpose of the study. If the short form is aimed at recognizing people with low levels of physical function and checking the relationship between low physical function and other long-term outcomes (such as frailty), then a lack of differences at higher levels of physical functioning is acceptable (39). The performance in these domains by patients who do not exhibit symptoms of depression, anxiety, or fatigue resembles that in the general population (40).

The initial seven-factor structure of the PROMIS-29 was supported by the CFA results. The model fit for sleep disturbances was not robust, which has also been found in prior studies (24). In this form, questions regarding “sleep quality” and “refreshment of sleep” could have some measurement error. Similar findings were reported by Huang et al. and Kang et al. (9, 24). Respondent bias in the interpretation of items or an overlap between these two items might have led to these errors. During the cognitive interview stage, we found that patients have difficulty understanding sleep issues. Sleep issues were also reflected in DIF. More research is needed to understand these effects better.

Moderate or strong correlations between the PROMIS-29 and the measures of similar constructs supported convergent validity. Additionally, discriminant validity was confirmed by negligible correlations between the PROMIS-29 and measures of dissimilar constructs. The five-symptom domains of the PROMIS-29 showed a strong correlation with similar concept legacy PRO measures (> 0.6), supporting the use of the PROMIS-29 questionnaire as a symptom assessment scale. Existing researches on PROs or QOL in older adults with chronic diseases frequently use the SF-36 and EQ-5D as measurement tools. The PROMIS-29 profile directly measures important symptoms, for instance, sleep problems, fatigue, anxiety, and depression, significantly reducing the measurement burden on patients (8, 24) compared to traditional PROs. Thus, the PROMIS-29 questionnaire may be more suitable for this patient population.

The results obtained from the questionnaire are reliable as determined by Cronbach’s *α* coefficient ≥ 0.80. These results have found a high Cronbach’s α coefficient for PROMIS profile domains, consistent with other studies (10, 24).

IRT-based analysis could provide more comprehensive item-level psychometric properties than the CTT. An analysis of the Rasch model showed that the domains satisfied the monotonic increasing assumption, confirming the unidimensional local independence of the seven domains with acceptable levels of reliability.

The PROMIS-29 profile is not reported to have measurement bias across individuals who differ in gender, age, and education (41). The study found that prominent DIF existed in different age groups for two items from the sleep disturbance domain, which was not reported in the study in the Dutch population with chronic diseases (10). Due to evolving sleep patterns and expectations with advancing age (42), there might be an item and concept understanding difference between community-dwelling older adults with multimorbidities of under and over age of 70 years. A previous study that analyzed the PROMIS-29 profile in older people with multiple chronic diseases found that only a small number of item pairs demonstrated statistically significant DIF, and all had negligible effect sizes (8). It is crucial to emphasize that the mere statistical significance of DIF does not necessarily equate to practical or clinical significance in the overall score interpretation. Two DIFs could be deemed acceptable. Consequently, we recommend retaining both items in the Chinese PROMIS-29. The instrument, as a whole, provided unbiased results across gender, age, and education groups for the vast majority of its items, supporting its overall fairness for use in our target population. Future studies with larger sample sizes could further monitor the performance of these items.

### 4.1 Limitations

This study has some limitations. First, our study findings are not generalizable because it was limited to convenience sampling and a population of community-dwelling older adults with multimorbidities from Central China only. Secondly, due to the cross-sectional design, the researchers did not assess the reactivity and interpretability of different clinical states. Additionally, due to the burden on the respondents, we did not compare PROMIS-29 with more commonly used PRO tools, such as EQ-5D or SF-36. Despite these limitations, the results of this study support the validity of the Chinese version of the PROMIS-29 (v2.1) profile for use in community-dwelling older adults with multimorbidities.

## 5 Conclusion

This study applied PROMIS-29 to the Chinese community-dwelling older individuals with multimorbidities earlier, providing an internationally standardized measurement tool for the health assessment of older comorbidities in Chinese communities, which is helpful for future research comparisons between populations in different regions of the world. As the use of the PROMIS-29 questionnaire increases, its use in primary care for HRQoL measurement in older adults with multimorbidities would serve as a valid and reliable tool. More applications, such as the development of computerized adaptive testing of the measures and electronic aging-friendly designs, need to be developed for use in community-dwelling older adults with chronic diseases. Future research may include direct comparisons with widely used measurement tools to further determine the relative utility of the Chinese version of the PROMIS-29 profile. In addition, with the PROMIS-29 scale incorporated into community chronic disease management, collecting multimodal data from physiological, psychological, and social health dimensions of patients can report their health changes to the system as soon as possible, achieving accurate evaluation and diagnosis of follow-up needs.

## Data Availability

The original contributions presented in the study are included in the article/Supplementary material, further inquiries can be directed to the corresponding author.

## Footnotes

^1^ http://www.healthmeasures.net

## Glossary

PROMIS-29: The patient-reported outcomes measurement information system 29-item profile
CIRF: The cognitive interviewing reporting framework
HRQoL: Health-related quality of life
SF-12: Short form-12 health survey
SSS-8: 8-Item somatic symptom scale
GAD-2: Generalized anxiety disorder-2
PHQ-2: Patient health questionnaire-2
CFA: Confirmatory factor analysis
PROs: Patient reported outcomes
PF: Physical functioning
SF: Social functioning
PCS: Physical component summary
MCS: Mental component summary
CFI: Comparative fit index
SRMR: Standardized root-mean-squared residual
RMSEA: Root mean square error of approximation
AIC: Akaike’s information criterion
IRT: Item response theory
ICC: Item characteristic curve
FCI: Functional comorbidity index
DIF: Differential item functioning
