# Correction: Psychometric validation of the Chinese version of the PROMIS-29 profile in community-dwelling older adults with multimorbidities

**DOI:** 10.3389/fpubh.2026.1779754

**Published:** 2026-01-30

**Authors:** Ting Zhao, Yan Zhang, Qinghua Cui, Min Zhang, Xiaoxia Han, Jialin Chen

**Affiliations:** 1 School of Nursing and Health, Zhengzhou University, Zhengzhou, Henan, China; 2 The First Affiliated Hospital of Zhengzhou University, Zhengzhou, Henan, China; 3 The Fifth Affiliated Hospital of Zhengzhou University, Zhengzhou, Henan, China; 4 School of Nursing, Fudan University, Shanghai, China

**Keywords:** PROMIS-29, psychometric validation, multimorbidity, older adults, item response theory

For author Ting Zhao, the order of the two affiliations was listed incorrectly in the original publication as:

1. The First Affiliated Hospital of Zhengzhou University, Zhengzhou, Henan, China

2. School of Nursing and Health, Zhengzhou University, Zhengzhou, Henan, China

The correct order should be:

1. School of Nursing and Health, Zhengzhou University, Zhengzhou, Henan, China

2. The First Affiliated Hospital of Zhengzhou University, Zhengzhou, Henan, China

Author “Huiping Xu” was erroneously included as an author in the published article. The correct author list reads:

Ting Zhao^1, 2^, Yan Zhang^* 1^, Qinghua Cui^1^, Min Zhang^2^, Xiaoxia Han^3^, Jialin Chen^4^

1 School of Nursing and Health, Zhengzhou University, Zhengzhou, Henan, China

2 The First Affiliated Hospital of Zhengzhou University, Zhengzhou, Henan, China

3 The Fifth Affiliated Hospital of Zhengzhou University, Zhengzhou, Henan, China

4 School of Nursing, Fudan University, Shanghai, China

The Author Contributions Statement has been corrected to read:

“TZ: Data curation, Investigation, Project administration, Software, Validation, Writing – original draft, Writing – review & editing. YZ: Conceptualization, Resources, Supervision, Funding acquisition, Writing – review & editing. QC: Investigation, Writing – review & editing. MZ: Conceptualization, Resources, Writing – review & editing. XH: Investigation, Resources, Writing – review & editing. JC: Project administration, Writing – review & editing.”

Author Huiping Xu was erroneously assigned as corresponding author. The correct corresponding author is Yan Zhang (zhangyanmy@126.com).

An incorrect Funding statement was provided.

The correct funder is the 2024 Central Plains Talent Program and the Research Topic of the Henan Provincial Federation of Social Sciences in 2023 (GKL-2023-569).

The Funding statement was erroneously given as “The author(s) declare that financial support was received for the research and/or publication of this article. This research was supported by the Nursing Special Project of the First Affiliated Hospital of Zhengzhou University (HLKY2023009) and Research Topic of the Henan Provincial Federation of Social Sciences in 2023 (GKL-2023-569).”

The correct funding statement reads:

“The author(s) declared that financial support was received for this work and/or its publication. This research was supported by the Research Topic of the Henan Provincial Federation of Social Sciences in 2023 (GKL-2023-569) and the 2024 Central Plains Talent Program (Educational Talent Series) Education and Teaching Leadership Fund.”

The original version of this article has been updated.

