# Supplementary material for: Psychometric validation of the Chinese version of the PROMIS-29 profile in community-dwelling older adults with multimorbidities
Source: Front Public Health. 2025 Oct 28;13:1631442. doi: 10.3389/fpubh.2025.1631442 (PMC12602215; doi:10.3389/fpubh.2025.1631442)
Supplement: Supplementary file 1 [file Table_1.DOCX]

Additional file 1: Known-group analysis results of PROMIS-29

|  | Gender [Mean(SD)] | | | | Age [Mean(SD)] | | | | | | Education [Mean(SD)] | | | | FCI score [Mean(SD)] | | | |
| --- | --- | --- | --- | --- | --- | --- | --- | --- | --- | --- | --- | --- | --- | --- | --- | --- | --- | --- |
| T-scores | male | \|d\| | female | P | 60~69 | \|d\| | 70~79 | \|d\| | ≥80 | P | Junior high school and below | \|d\| | High school and above | P | ≤3 | \|d\| | ≥4 | p |
| Physical function | 48.9（9.0） | 0.0 | 48.9（8.5） | 0.825 | 52.0(7.0) | 3.5 | 48.5(8.5) | 3.8 | 44.7（9.6） | ＜0.001 | 48.3（8.9） | 1.5 | 49.8（8.3） | 0.035 | 50.1（8.3） | 3.4 | 46.7（9.0） | ＜0.001 |
| Anxiety | 47.4（8.2） | 2.3 | 49.2(9.1) | ＜0.001 | 49.2(9.1) | 1.0 | 48.2(8.4) | 0.7 | 48.9（9.0） | 0.610 | 48.2（8.9） | 1.1 | 49.3（8.5） | 0.125 | 47.4（7.9） | 3.6 | 51.0（9.7） | ＜0.001 |
| Depression | 47.2(7.7) | 2.3 | 49.5(8.2) | ＜0.001 | 48.0(8.8) | 0.2 | 48.2(7.6) | 1.5 | 49.7（7.9） | 0.091 | 49.0（8.4） | 1.4 | 47.6（7.5） | 0.042 | 47.4（7.4） | 7.1 | 54.5（9.0） | ＜0.001 |
| Fatigue | 44.7(9.2) | 2.4 | 47.1(9.6) | 0.002 | 45.6(9.0) | 0.5 | 46.1(9.5） | 0.3 | 46.4（10.5） | 0.762 | 46.3（9.9） | 0.7 | 45.6（8.9） | 0.553 | 44.2（9.4） | 5.1 | 49.3（8.8） | ＜0.001 |
| Sleep disturbance | 48.6(8.2) | 3.9 | 52.5(8.7) | ＜0.001 | 50.4（8.8） | 0.5 | 50.9（8.4） | 0.0 | 50.9（9.1） | 0.579 | 50.9（9.0） | 0.4 | 50.5（8.2） | 0.918 | 50.0（8.7） | 2.1 | 52.1（8.5） | 0.002 |
| Social roles | 57.5(8.6) | 1.9 | 55.6(8.4) | 0.001 | 58.1（8.0） | 1.6 | 56.5（8.3） | 2.8 | 53.7（9.3） | ＜0.001 | 56.3(8.6) | 0.5 | 56.8（8.4） | 0.458 | 57.6（8.1） | 3.1 | 54.5（9.0） | ＜0.001 |
| Pain interference | 48.9（8.2) | 2.6 | 51.5(8.5) | ＜0.001 | 49.8（8.0） | 0.7 | 50.5（8.5） | 0.4 | 50.9（8.9） | 0.599 | 50.7（8.7） | 0.9 | 49.8（8.0） | 0.336 | 48.6（7.8） | 4.8 | 53.4（8.6） | ＜0.001 |
